# Supplementary material for: Prospect of acromegaly therapy: molecular mechanism of clinical drugs octreotide and paltusotine
Source: Nat Commun. 2023 Feb 21;14:962. doi: 10.1038/s41467-023-36673-z (PMC9944328; doi:10.1038/s41467-023-36673-z)
Supplement: Supplementary file 1 — Supplementary Information [file 41467_2023_36673_MOESM1_ESM.pdf]

Prospect of acromegaly therapy: molecular mechanism of clinical drugs octreotide and paltusotine

Supplementary information

Supplementary Figures 1-10
Supplementary Tables 1-7

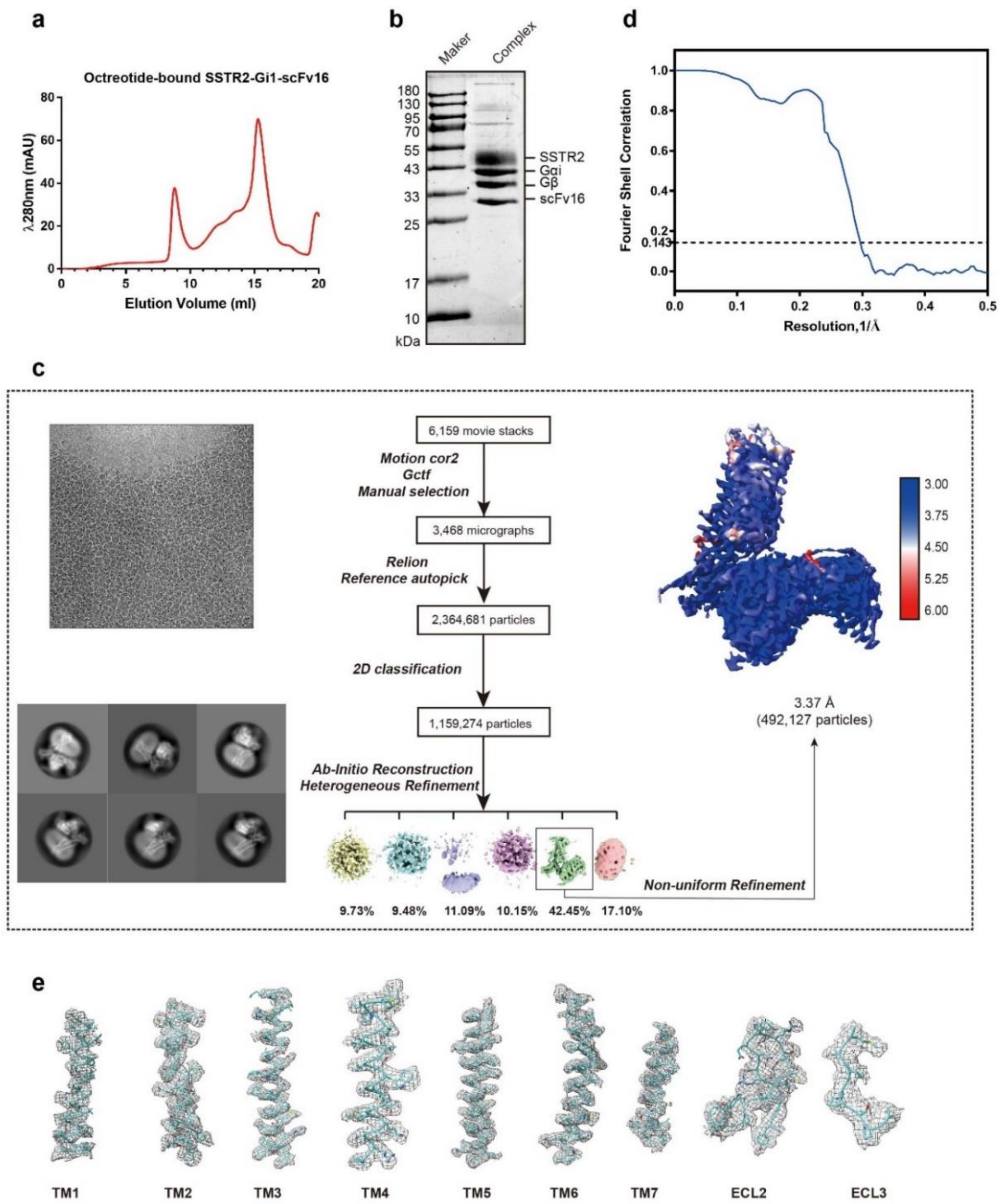

Supplementary Fig. 1: Octreotide-bound SSTR2-Gα_{i1}-G$\beta_1\gamma_2$-scFv16 complex purification, cryo-EM data collection and structural determination.

a, Size-exclusion chromatography results of the purified octreotide-bound SSTR2-Gα_{i1}-G$\beta_1\gamma_2$-scFv16 complex.

b, The representative SDS-PAGE analysis of purified octreotide-bound SSTR2-Gα_{i1}-G$\beta_1\gamma_2$-scFv16 protein. Data is repeated over three times.

c, Flow chart of the cryo-EM data processing of octreotide-bound SSTR2-Gα_{i1}-G$\beta_1\gamma_2$-scFv16 complex, including particle selection, classifications and density map reconstruction.

d, The gold-standard Fourier shell correlation (FSC) curves of octreotide-bound SSTR2-Gα_{i1}-G$\beta_1\gamma_2$-scFv16 complex, which shows a global resolution of 3.37 Å according to the FSC=0.143 cut off.

e, Representative cryo-EM density maps and structural models for all seven transmembrane α-helices, as well as the extracellular loop 2 and 3.

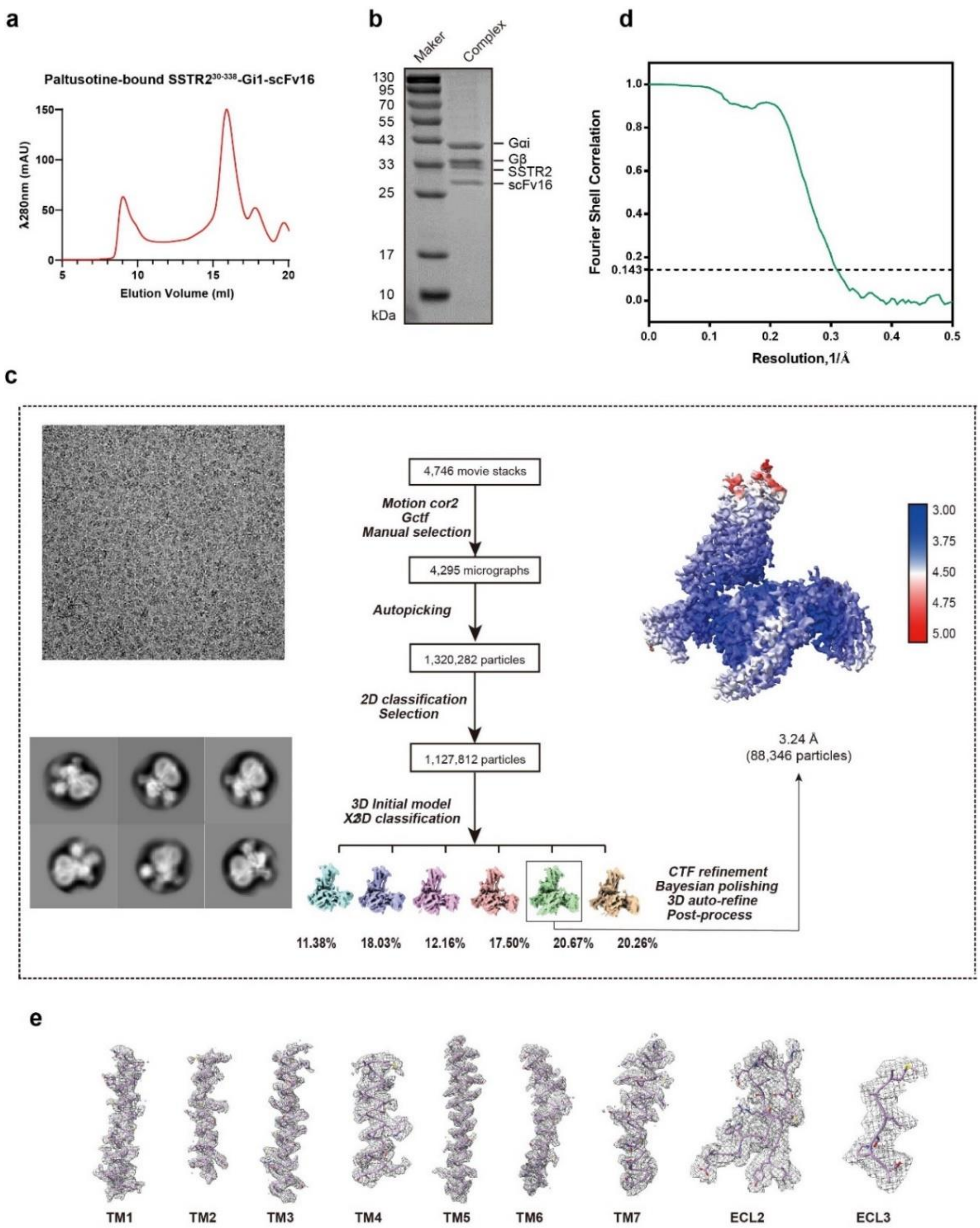

Supplementary Fig. 2: Paltusotine-bound SSTR2-Gα_{i1}-G$\beta_1\gamma_2$-scFv16 complex purification, cryo-EM data collection and structural determination.

a, Size-exclusion chromatography results of the purified paltusotine-bound SSTR2-Gα_{i1}-G$\beta_1\gamma_2$-scFv16 complex.

b, The representative SDS-PAGE analysis of purified paltusotine-bound SSTR2-Gα_{i1}-G$\beta_1\gamma_2$-scFv16 protein. Data is repeated over three times.

c, Flow chart of the cryo-EM data processing of paltusotine-bound SSTR2-Gα_{i1}-G$\beta_1\gamma_2$-scFv16 complex, including particle selection, classifications and density map reconstruction.

d, The gold-standard Fourier shell correlation (FSC) curves of paltusotine-bound SSTR2-Gα_{i1}-G$\beta_1\gamma_2$-scFv16 complex, which shows a global resolution of 3.24 Å according to the FSC=0.143 cut off.

e, Representative cryo-EM density maps and structural models for all seven transmembrane α-helices, as well as the extracellular loop 2 and 3.

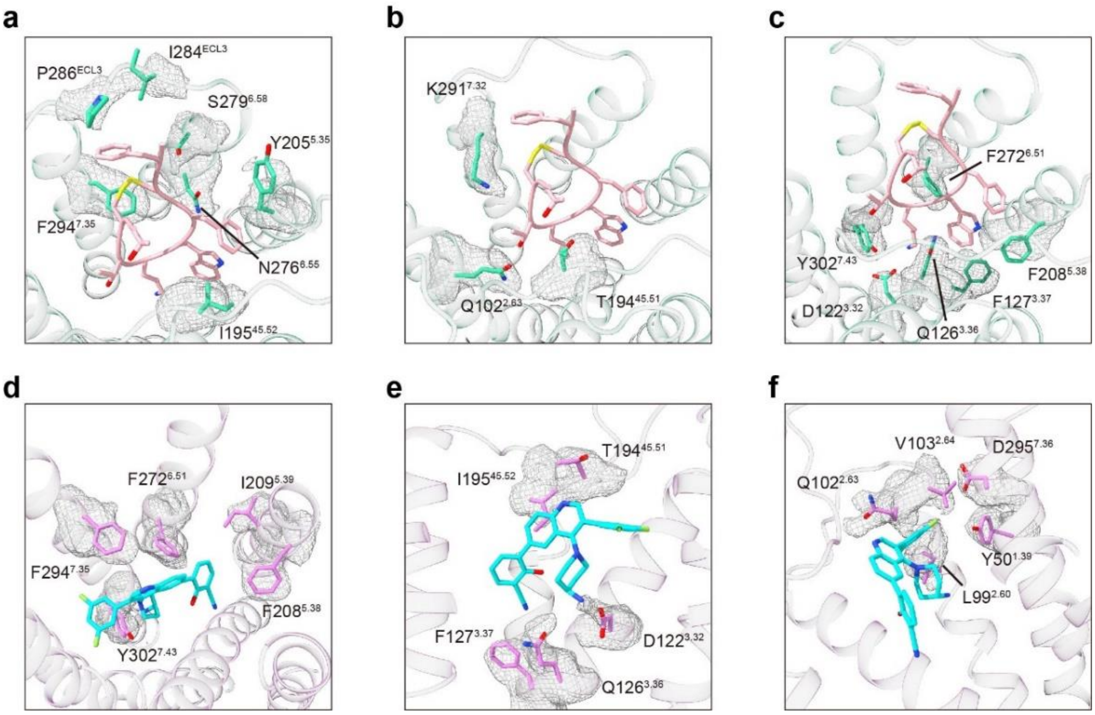

Supplementary Fig. 3: The electron density map of the key residues involved in ligand binding.

a-c, The electron density map of the residues in the octreotide binding pocket. SSTR2 is shown in a cartoon diagram and colored in green-cyan; octreotide is shown as sticks and colored in light pink; density map is shown in gray meshes at contour level of 0.4.

d-f, The electron density map of the residues in the paltusotine binding pocket. SSTR2 is shown in a cartoon diagram and colored in violet; paltusotine is shown as sticks and colored in cyan; density map is shown in gray meshes at contour level of 0.004.

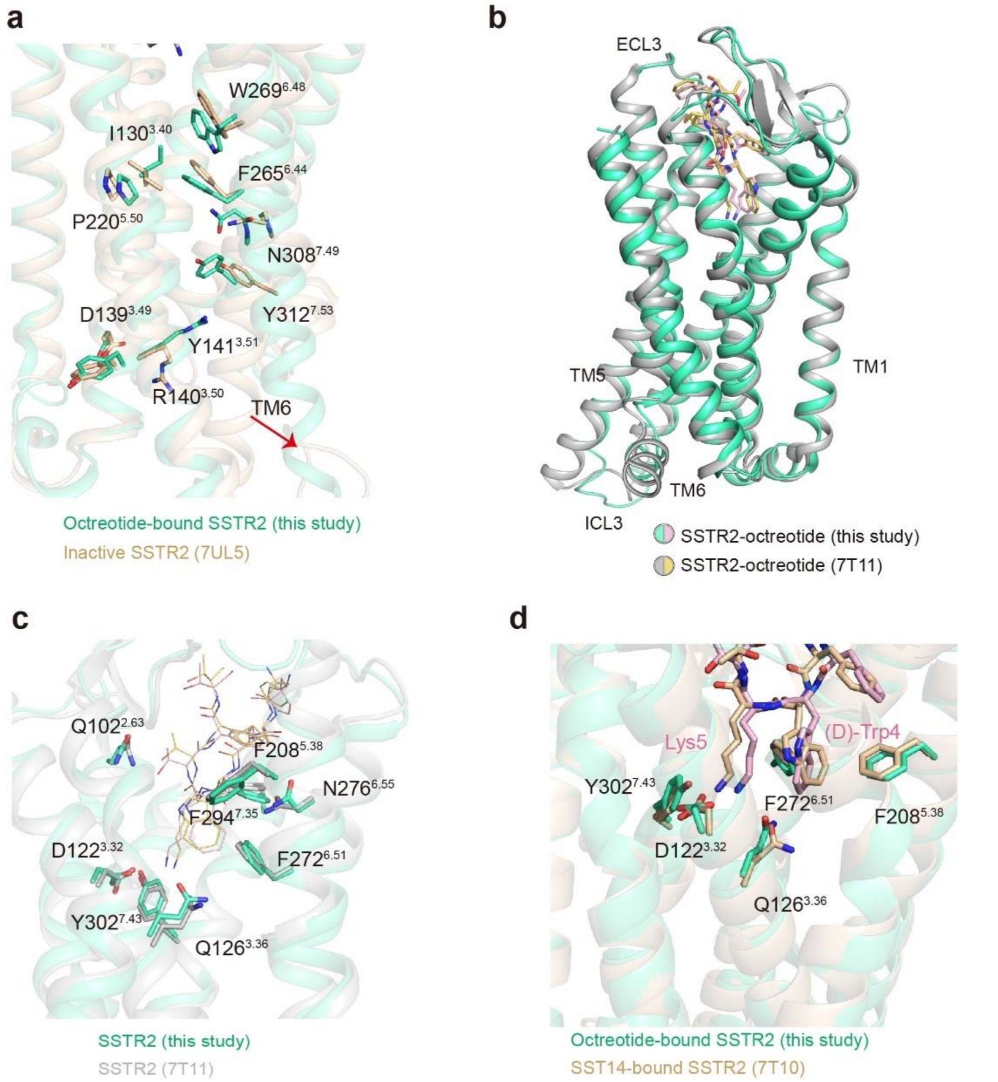

Supplementary Fig. 4: Structural comparison of SSTR2-octreotide with previously reported structures.

a, Structural alignment of SSTR2-octreotide structure solved by this study (colored in green-cyan) with the inactive SSTR2 structure (PDB code: 7UL5, colored in wheat). Microswitch residues are shown as sticks.

b, c, Structural comparison of SSTR2-octreotide structures determined by previous studies and this study. **(b)** Overall structural comparison of SSTR2-octreotide in our study with SSTR2[7T11]. SSTR2 in our study is shown as cartoon and colored in green-cyan, octreotide is shown as sticks and colored in light pink; SSTR2 in SSTR2[7T11] is shown as cartoon and colored in gray, octreotide is shown as sticks and colored in yellow. **(c)** Structural comparison of the residues in the octreotide binding pocket of SSTR2 in these two structures. Key residues in SSTR2 are shown as sticks.

d, Structural alignment of SSTR2-octreotide structure (colored in green-cyan) SST14-bound SSTR2 structure (PDB code: 7T10, colored in wheat). Key residues are shown as sticks.

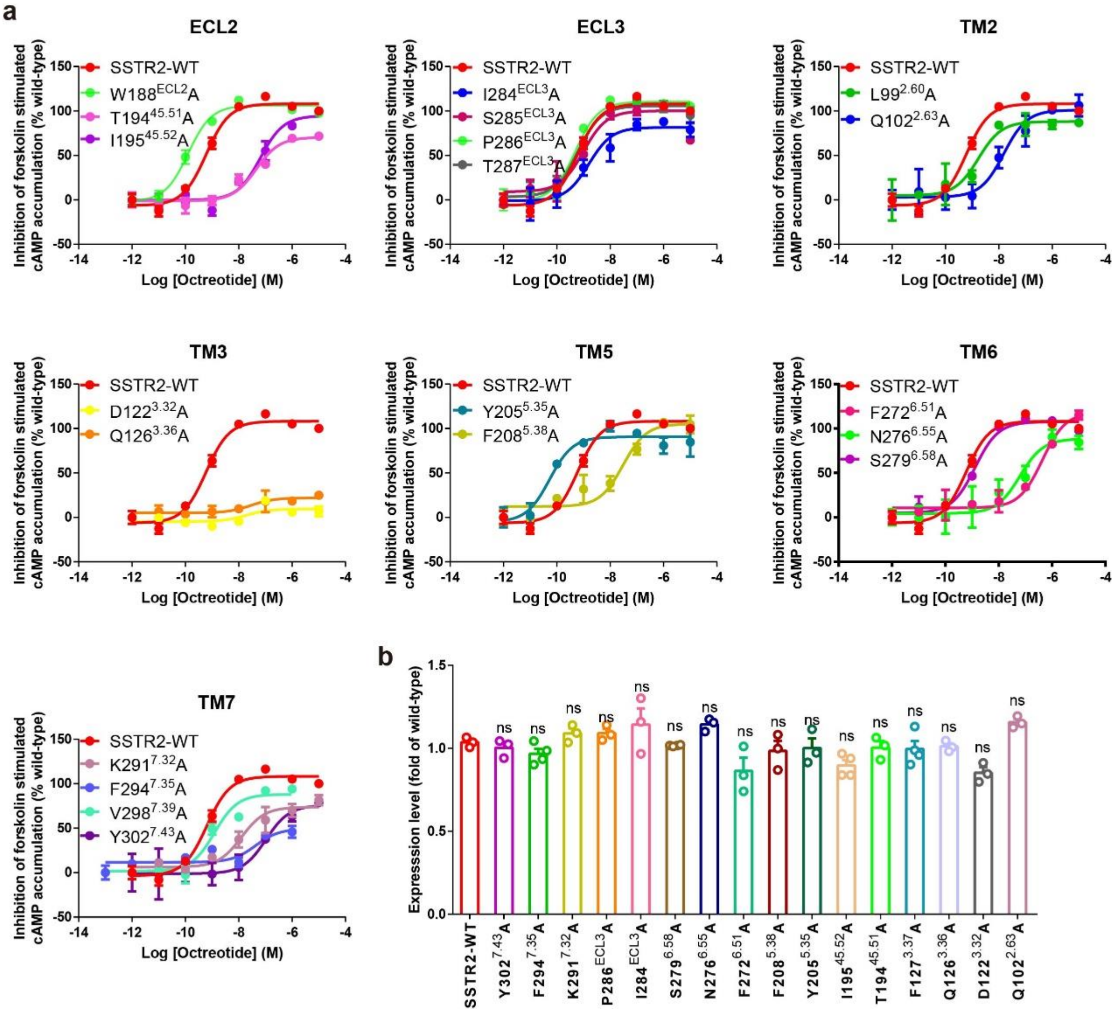

Supplementary Fig. 5: The effects of the substitution of the SSTR2 residues within the octreotide binding pocket to Gi signaling transduction.

a, Representative effects of the residue substitution in TM2, TM3, TM5, TM6, TM7, ECL2 and ECL3 of SSTR2 on the agonist octreotide induced cAMP inhibition assays. Data represent mean ± SEM from three independent experiments for wild-type SSTR2 and its mutants.

b, The expression level of the wild-type SSTR2 and the SSTR2 mutants measured by detecting the cell surface expression by elisa assays. Statistical differences between wild-type and mutants were determined by one-way of variance ANOVA with Dunnett's test. n.s., not significant (P =0.9994, 0.9705, 0.3804, 0.9993, 0.9956, 0.7656, 0.9996, 0.7553, 0.1740, 0.9959, 0.9993, 0.9994, 0.9991, 0.9996, 0.1531, 0.6255 from left to right). Data represent mean ± SEM from three independent experiments for wild-type SSTR2 and its mutants.

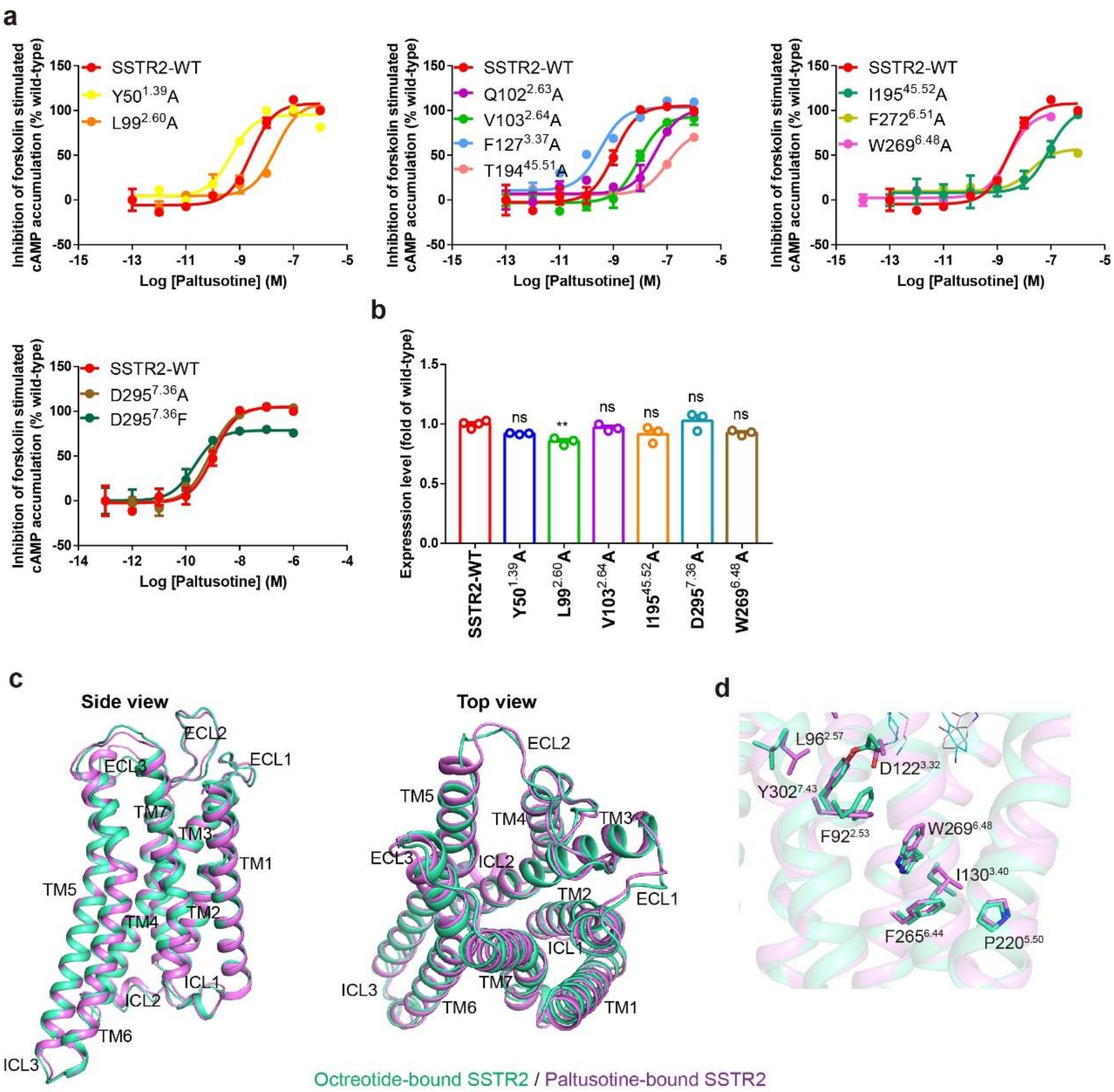

Supplementary Fig. 6: The binding pocket of paltusotine, and the effects of the substitution of the SSTR2 residues within the paltusotine binding pocket to Gi signaling transduction.

a, Representative effects of the substitution of the residues within the paltusotine binding pocket of SSTR2 on the paltusotine-induced cAMP inhibition assays. Data represent mean ± SEM from three independent experiments for wild-type SSTR2 and its mutants.

b, The expression level of the wild-type SSTR2 and its mutants measured by detecting the surface expression by elisa assays. Statistical differences between wild-type and mutants were determined by one-way of variance ANOVA with Dunnett's test. $^{**}P < 0.002$; n.s., not significant ($P = 0.1675$, 0.0034, 0.9199, 0.1302, 0.9591, 0.2388 from left to right). Data represent mean ± SEM from three independent experiments for wild-type SSTR2 and its mutants.

c, The overall structural comparation of SSTR2 in the octreotide- or paltusotine-bound complex. SSTR2 is shown in cartoon, with the octreotide-bound SSTR2 colored in green-cyan and the paltusotine-bound SSTR2 colored in violet.

d, The downstream F92$^{2.53}$, L96$^{2.57}$, D122$^{3.32}$, Y302$^{7.43}$ and the microswitches W269$^{6.48}$ and P220$^{5.50}$-I130$^{3.40}$-F265$^{6.44}$ motif are shown in sticks, which exhibit obvious conformational change in these two complexes.

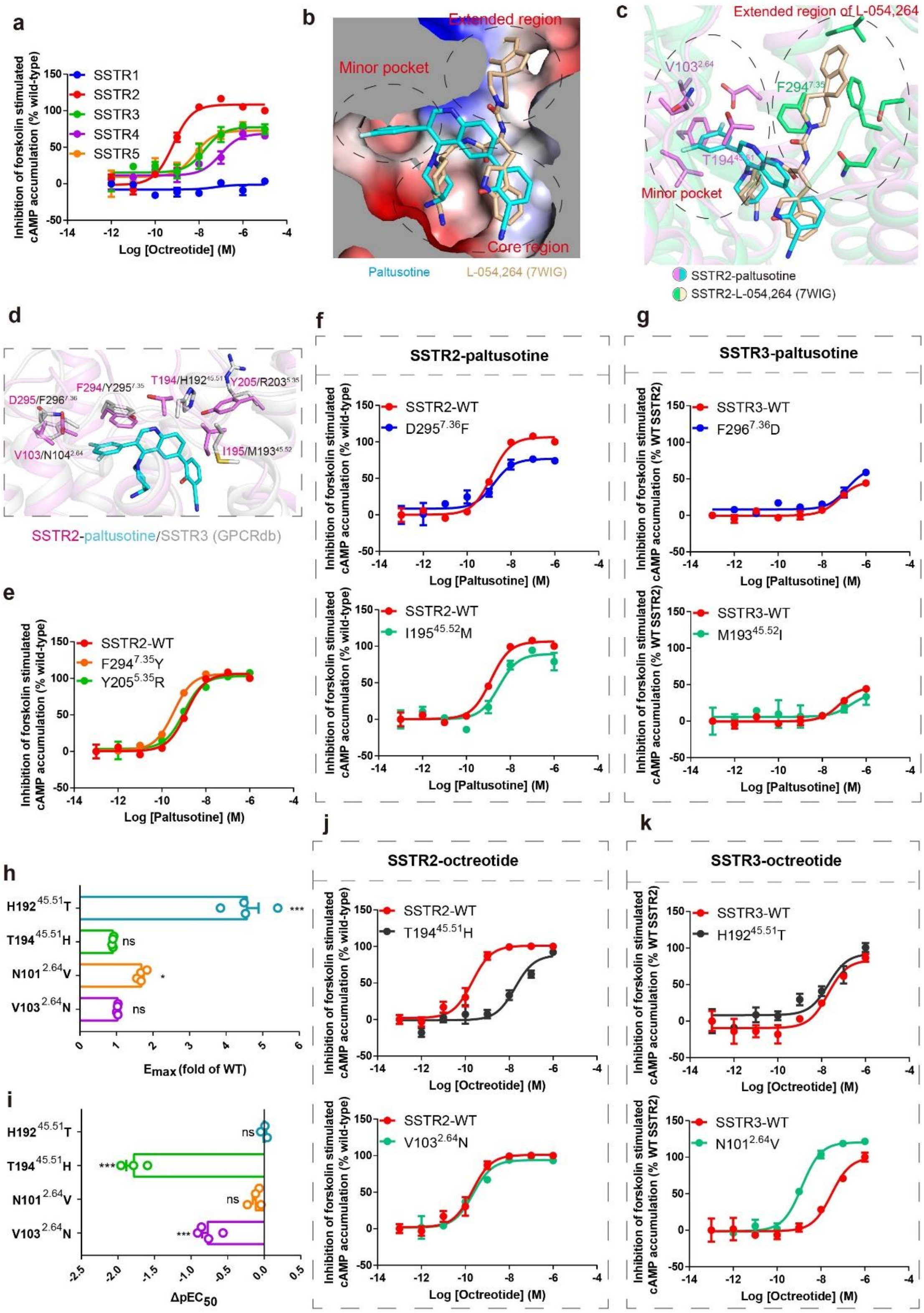

Supplementary Fig. 7: The ligand selectivity analysis of paltusotine on SSTR2 among other group 2 subtypes compared with octreotide.

a, The Gi signaling of SSTR1-5 activated by octreotide. Data represent mean ± SEM from three independent experiments.

b, Structural comparison of paltusotine (cyan), L-054,264^{7WIG} (wheat). Small molecules are shown as sticks and SSTR2 is shown in an electrostatic surface representation.

c, Structural comparison of SSTR2-paltusotine and SSTR2-L-054,264^{7WIG}. SSTR2 in our study is shown as cartoon and colored in violet, paltusoitne is shown as sticks and colored in cyan. SSTR2 in SSTR2-L-054,264^{7WIG} is shown as cartoon and colored in lime green, L-054,264 is shown as sticks and colored in wheat. Key residues involving in ligand binding in SSTR2 are shown as sticks, residues only crucial for paltusotine binding are labeled in violet; residues only involve in L-054,264 binding are labeled in wheat.

d, The three-dimensional structural comparison of the indicated residues in SSTR2 and SSTR3 in (**4b**). Residues are shown in sticks and colored in violet (paltusotine-bound SSTR2) and gray (active form of SSTR3 predicted by GPCRdb), with paltusotine colored in cyan.

e, The response curves of the SSTR2 mutants which have no significant effects on the paltusotine-induced Gi signal compared to wild-type. Data represent mean ± SEM from three independent experiments for wild-type SSTR2 and its mutants.

f, g, The effects of the residues substitution of SSTR2 with SSTR3 (**f**), or the residues substitution of SSTR3 with SSTR2 (**g**) on the paltusotine-induced cAMP inhibition. Data represent mean ± SEM from three independent experiments for wild-type SSTRs and its mutants.

h, i, The E_{max} (**h**, expressed as the fold of that in the corresponding WT SSTR2 or SSTR3) and the ΔpEC_{50} (**i**) of the mutants in (**4c**) and (**4d**). Statistical differences between wild-type and mutants were determined by one-way of variance ANOVA with Dunnett's test. *P < 0.33; ***P < 0.001; ns, not significant. E_{max} (P=<0.001, 0.979, 0.034, >0.999, from top to bottom). ΔpEC_{50} (P=0.519, <0.001, 0.275, <0.001, from top to bottom). Data represent mean ± SEM from three independent experiments.

j, k, The effects of the residues substitution of SSTR2 with SSTR3 (**j**), or the residues substitution of SSTR3 with SSTR2 (**k**) on the octreotide-induced cAMP inhibition corresponding to **Fig. 4c** and **4d**. Data represent mean ± SEM from three independent experiments for wild-type SSTRs and its mutants.

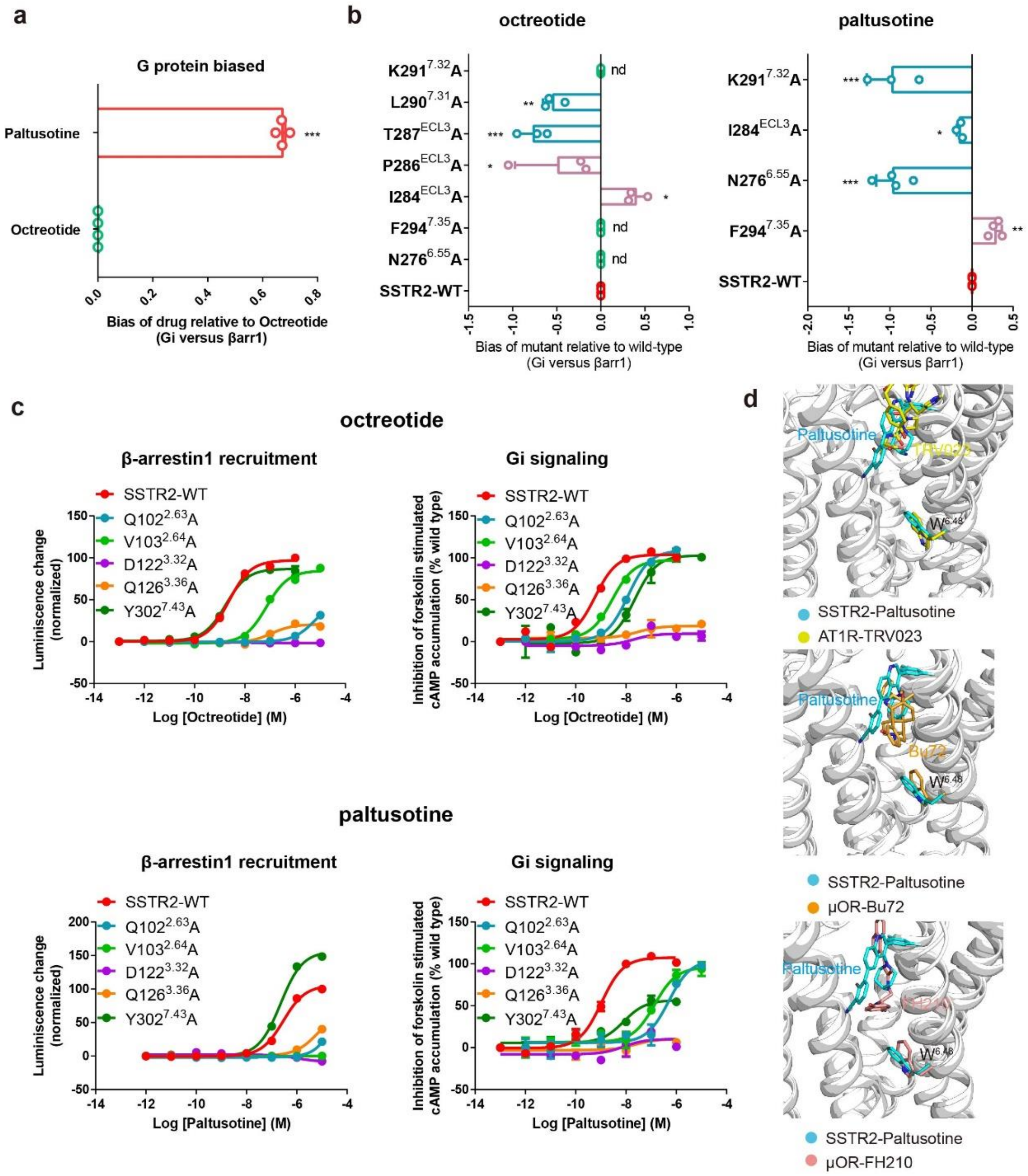

Supplementary Fig. 8: Biased property analyses of the residues within the octreotide or paltusotine binding pocket in SSTR2.

a, Bias factors of paltusotine relative to octreotide calculated by Gi signal assay response curves and arrestin recruitment response curves. Statistical differences between octreotide and paltusotine were determined by one-way of variance ANOVA with T- test (P=<0.001). ***p < 0.001. Data represent mean ± SEM from three independent experiments.

b, Bias factors of the substitution of the residues within the octreotide extended binding pocket induced by octreotide (left) and paltusotine (right). Statistical differences between wild-type and mutants were determined by one-way of variance ANOVA with Dunnett's test. ***p < 0.001, **p < 0.002, *p < 0.033, n.s, not significant; n.d., not detected. Octreotide (P = n.d., <0.002, <0.001, <0.033, <0.033., n.d., n.d., from top to bottom). Paltusotine (P =<0.001, <0.033, <0.001, n.d., from top to bottom). Data represent mean ± SEM from three independent experiments. β value >0 denotes the mutant affects β-arrestin recruitment more than Gi signaling activation.

c, The effects of SSTR2 mutations on cAMP inhibition and β-arrestin recruitment induced by octreotide and paltusotine. Data represent mean ± SEM from three independent experiments.

d, The binding pocket of biased ligand TRV023 (yellow, PDB code: 6OS1) in AT1R, agonist Bu72 (bright orange, PDB code: 5C1M) or FH210 (light pink, PDB code: 7SCG) in μOR compared with paltusotine (blue) in SSTR2. The downstream microswitch $W^{6.48}$ is shown in sticks and colored in the same color with its corresponding ligand.

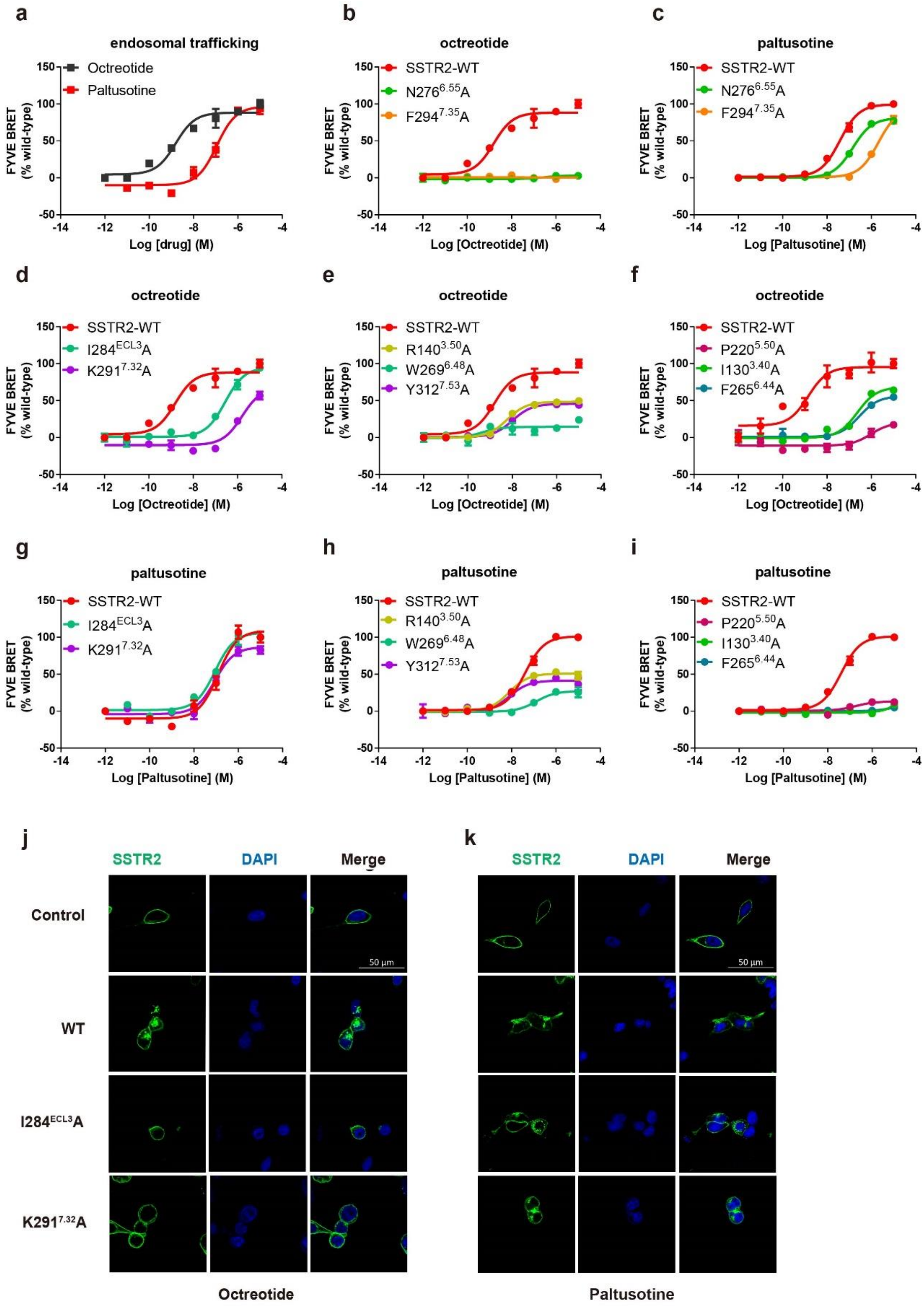

Supplementary Fig. 9: Differential internalization activated by octreotide and paltusotine measured by Bystander BRET assays.

a, Internalization of SSTR2 in HEK293 cells treated with octreotide and paltusotine measured by Bystander BRET assays in the presence of the early endosome marker FYVE. Data represent mean ± SEM from three independent experiments.

b, c, Effects of N276$^{6.55}$A or F294$^{7.35}$A mutation on the internalization of SSTR2 in HEK293 cells treated with octreotide (**b**) or paltusotine (**c**) indicated by Bystander BRET assays in the presence of the early endosome marker FYVE. Data represent mean ± SEM from three independent experiments.
d-f, Effects of I284^{ECL3}A, K291$^{7.32}$A (**d**) or microswitch residues (**e, f**) mutation on the internalization of SSTR2 in HEK293 cells treated with octreotide in the presence of the early endosome marker FYVE. Data represent mean ± SEM from three independent experiments.
g-i, Effects of I284^{ECL3}A, K291$^{7.32}$A (**g**) or microswitch residues (**h, i**) mutation on the internalization of SSTR2 in HEK293 cells treated with paltusotine in the presence of the early endosome marker FYVE. Data represent mean ± SEM from three independent experiments.
j, k, Effects of I284^{ECL3}A or K291$^{7.32}$A mutation on the internalization of SSTR2 in HEK293 cells treated with octreotide (**j**) and paltusotine (**k**) indicated by confocal fluorescence microscopy. eGFP-tagged SSTR2 wild-type or mutant plasmid was transfected in HEK293 cells and then cells were treated with 10 μM octreotide or paltusotine for 30 min and analyzed by confocal fluorescence microscopy (green, eGFP-SSTR2; blue, DAPI). Scale bar, 50 μm.

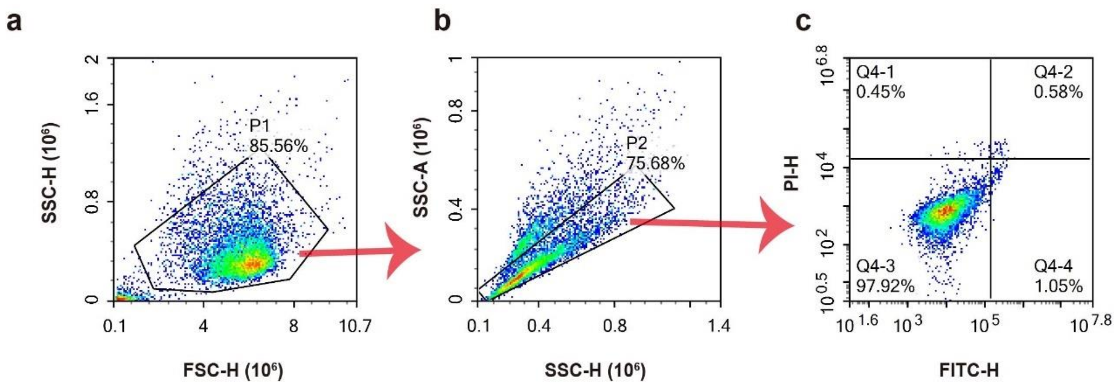

Supplementary Fig. 10: Gating strategy and flow cytometry analysis of apoptotic cells.

The example of gating strategy for analysis of the apoptotic cells is from the control group in **Fig.7a**. (**a)** The P1 gate in forward and side scatter were gated to get the population of cells; (**b)** P2 in area versus height side scatter were gated to select the single cells; (**c)** cells in Q4-4 (Annexin V+/PI−) and Q4-2 (Annexin V+/PI+) gates were both considered as the apoptotic cells. The data panel in (**c**) is corresponded to the panel in **Fig.7a**.

Supplementary Table 1. The cryo-EM data collection, refinement and validation statistics.

	**Octreotide-bound SSTR2-Gi**	**Paltusotine-bound SSTR2-Gi**
**Data collection and processing**		
Magnification	130,000	165,000
Voltage (keV)	300	300
Electron exposure ($e^-/Å^2$)	65	65
Defocus range (μm)	-2.0~-1.0	-2.0~-1.0
Pixel size (Å)	0.46	0.85
Symmetry imposed	C1	C1
Initial particle projections (no.)	2,364,681	1,320,282,
Final particle projections (no.)	492,127	88,346
Map resolution (Å)	3.37	3.24
FSC threshold	0.143	0.143
Map resolution range (Å)	3.0~6.0	3.0~6.8
**Refinement**		
Model resolution (Å)	3.8	3.3
FSC threshold	0.5	0.5
Map sharpening *B* factor ($Å^2$)	-185.60	-85.45
**Model composition**		
Non-hydrogen atoms	9031	8937
Protein residues	1140	1140
Ligand	1	1
Lipid	2	0
***B* factors ($Å^2$)**		
Receptor	81.46	100.54
Ligand	94.65	107.18
**R.m.s deviations**		
Bond lengths (Å)	0.009	0.002
Bond angles (°)	0.784	0.573
**Validation**		
Molprobity score	1.97	1.60
Clashscore	12.57	6.12
**Ramachandran plot** [e]		
Most favored regions (%)	94.76	96.09
Allowed regions (%)	5.24	3.91
Outliers (%)	0.00	0.00

Supplementary Table 2. Summary of octreotide induced cAMP inhibition assay and NanoBiT β-arrestin 1 recruitment assay of SSTR2. Related to Figs. 2, 5 and Supplementary Figs. 5, 8.

Mutation	EC_{50} (nM)	Fold of WT	E_{max} (%WT)	n	EC_{50} (nM)	Fold of WT	E_{max} (%WT)	n	Expression Level (%WT)
	Gi signaling				β-arrestin recruitment				
WT	0.46±1.19	1	100	6	3.54±4.12	1	100	6	100
$I284^{ECL3}A$	1.91±4.66	4.20	97.53±14.28	3	55.70±2.03	15.73	89.03±9.00	3	114.20 ±9.70
$S285^{ECL3}A$	0.69±1.18	1.50	87.52±6.00	3	0.84±5.03	1.83	79.09±5.45	3	92.32 ±4.70
$P286^{ECL3}A$	0.62±1.18	1.35	102.40±5.34	3	6.35±1.59	1.79	76.51±12.11	3	109.10 ±2.63
$W188^{ECL3}A$	0.32±2.21	0.69	102.40±5.12	3	4.34±9.50	1.23	69.46±6.21	3	112.30±4.59
$T287^{ECL3}A$	0.60±4.17	1.30	98.50±5.26	3	4.71±8.35	1.33	87.90±2.95	3	100.40±5.73
$T194^{45.51}A$	48.53±2.29	105.50	91.51±3.38	3	48.70±3.38	13.76	55.69±0.38	3	100.40±3.89
$I195^{45.52}A$	6.58±1.10	14.30	100.40±3.58	3	54.33±4.10	15.35	59.10±0.47	3	89.61±3.36
$Y50^{1.39}A$	0.46±2.12	1.00	104.70±0.48	3	4.87±3.17	1.38	61.91±5.26	3	91.28±0.76
$L99^{2.60}A$	1.25±5.55	2.72	92.36±6.12	3	91.60±3.34	25.88	96.13±25.27	3	116.80±5.09
$Q102^{2.63}A$	66.83±2.72	145.28	71.48±1.31	3	ND	ND	57.95±5.20*	3	98.81 ±7.68
$V103^{2.64}A$	7.66±2.51	16.65	90.80±3.48	3	63.67±5.11	17.99	84.83±0.26	3	89.22± 7.63
$D122^{3.32}A$	ND	ND	12.04±1.83*	3	ND	ND	ND	3	85.19 ±3.31
$Q126^{3.36}A$	ND	ND	21.88±1.91*	3	ND	ND	ND	3	101.10±1.88
$F127^{3.37}A$	1.93±6.77	4.20	108.70±0.91	3	4.33±1.53	1.22	52.62±2.57	3	-
$I130^{3.40}A$	11.62±4.99	25.26	101.10±5.08	3	ND	ND	76.52±9.52*	3	103.00±3.81
$Y205^{5.35}A$	0.92±3.96	2.00	112.90±25.77	3	8.52±5.74	2.40	108.30±13.91	3	100.10±5.93
$F208^{5.38}A$	6.42±2.37	13.96	96.46±2.20	3	163.30±6.17	46.13	75.93±9.81	3	98.34 ±6.18
$P220^{5.50}A$	3.37±1.32	7.33	95.86±14.17	3	99.85±9.00	28.20	61.16±16.24	3	103.80±2.36
$F265^{6.44}A$	12.26±5.46	26.65	99.81±4.55	3	ND	ND	67.17±9.75*	3	117.80±3.62
$W269^{6.48}A$	0.89±2.24	1.93	99.58±2.56	3	55.30±1.35	15.62	33.36±2.81	3	91.76±1.31
$F272^{6.51}A$	322.30±1.60	700.65	95.01±21.17	3	ND	ND	ND	3	85.67 ±8.42
$N276^{6.55}A$	35.13±8.45	76.37	107.20±5.70	3	ND	ND	ND	3	114.30±2.22
$S279^{6.58}A$	1.17±1.01	2.54	101.70±5.61	3	11.88±4.76	3.36	87.86±7.76	3	101.60±0.20
$L290^{7.31}A$	0.42±8.68	0.91	103.50±7.16	3	1.80±9.50	0.54	85.66±1.04	3	107.10±2.39
$K291^{7.32}A$	5.46±1.06	11.87	89.41±10.26	3	ND	ND	ND	3	117.30±8.66
$F294^{7.35}A$	190.3±2.07	413.70	96.11±5.83	3	ND	ND	ND	3	96.62±3.10
$D295^{7.36}A$	4.40±8.48	9.57	102.80±9.54	3	319.07±6.78	90.13	119.20±34.68*	3	110.80±8.60
$Y302^{7.43}A$	79.03±2.70	171.80	79.09±10.74	3	1.82±6.40	0.51	109.40±18.82	3	100.30 ±3.16

ND means not detected. "–" means no measurement.

"*" means detected at 10 μM.

Supplementary Table 3. Summary of paltusotine induced cAMP inhibition assay and NanoBiT β-arrestin 1 recruitment assay of SSTR2. Related to Figs. 3, 5 and Supplementary Figs. 6, 8.

Mutation	EC_{50} (nM)	Fold of WT	E_{max} (%WT)	n	EC_{50} (nM)	Fold of WT	E_{max} (%WT)	n	Expression Level (%WT)
	Gi signaling				β-arrestin recruitment				
**WT**	1.88±3.15	1	100	6	405.70±5.62	1	100	6	100
**I284^{ECL3}A**	1.22±4.26	0.80	113.80±5.04	3	180.70±3.33	0.45	192.70±19.45	3	114.20 ±9.70
**T194$^{45.51}$A**	97.73±2.20	51.98	85.51±3.13	3	ND	ND	95.9±9.02	3	100.40±3.892
**I195$^{45.52}$A**	83.40±1.65	44.36	106.20±8.71	3	ND	ND	ND	3	89.61±3.36
**Y50$^{1.39}$A**	3.60±6.86	1.90	106.10±0.90	3	68.73±2.87	0.17	90.74±11.02	3	91.28±0.76
**L99$^{2.60}$A**	17.09±5.16	9.09	107.80±2.34	3	ND	ND	84.26±3.71*	3	116.80 ±5.09
**Q102$^{2.63}$A**	31.03±6.59	16.51	97.26±5.45	3	ND	ND	26.07±1.00*	3	98.81 ±7.68
**V103$^{2.64}$A**	32.97±2.87	17.54	87.33±6.38	3	ND	ND	ND	3	89.22± 7.63
**D122$^{3.32}$A**	ND	ND	ND	3	ND	ND	ND	3	85.19 ±3.31
**Q126$^{3.36}$A**	ND	ND	ND	3	ND	ND	65.37±3.31*	3	101.10±1.88
**F127$^{3.37}$A**	1.02±1.29	0.54	107.70±2.32	3	198.00±6.66	0.49	60.24±1.11	3	-
**I130$^{3.40}$A**	18.97±2.64	10.09	116.50±4.76	3	ND	ND	30.45±0.36*	3	103.00±3.81
**Y205$^{5.35}$A**	0.48±2.23	0.26	98.71±2.78	3	171.70±2.07	0.42	152.00±23.85	3	100.10 ±5.93
**F208$^{5.38}$A**	64.23±2.53	34.16	91.78±2.89	3	ND	ND	70.89±2.39*	3	98.34 ±6.18
**P220$^{5.50}$A**	3.22±1.38	1.71	116.00±5.23	3	ND	ND	50.55±2.81*	4	103.80±2.36
**F265$^{6.44}$A**	11.94±1.45	6.35	113.70±4.57	3	ND	ND	49.61±1.04*	4	117.80±3.62
**W269$^{6.48}$A**	0.44±1.61	0.23	98.94±12.38	3	1077.00±6.28	2.65	50.80±24.99	3	91.28±0.76
**F272$^{5.65}$A**	63.13±4.10	33.58	57.21±2.64	3	ND	ND	ND	3	85.67 ±8.42
**N276$^{6.55}$A**	1.65±3.51	0.88	89.08±4.63	3	69.13±4.07	0.17	157.20±15.71	3	114.30±2.22
**K291$^{7.32}$A**	5.22±8.67	2.78	97.49±6.65	3	86.70±3.50	0.21	133.50±14.76	3	117.30±8.66
**F294$^{7.35}$A**	2.64±1.26	1.40	100.30±10.53	3	990.70±5.93	2.44	114.80±11.09	3	96.62±3.10
**D295$^{7.36}$A**	2.46±1.67	1.30	107.80±3.11	3	495.70±1.76	1.22	87.12±2.30*	3	110.80±8.60
**Y302$^{7.43}$A**	11.79±3.12	6.27	55.80±2.34	3	225.30±6.67	0.56	190.00±17.00	3	100.30 ±3.16

ND means not detected. "–" means no measurement.

" * "means detected at 10 μM.

Supplementary Table 4. Summary of paltusotine induced cAMP inhibition assays of SSTRs. Related to Fig. 4 and Supplementary Fig. 7.

Mutation	EC_{50} (nM)	Fold of WT	E_{max} (%WT SSTR2)	n	Expression level (%WT)
**SSTR2-WT**	2.08±1.39	1.00	100	6	100
**V103$^{2.64}$N**	11.96±1.33	5.75	99.52±4.73	3	101.30 ± 0.69
**T194$^{45.51}$H**	150.70±1.56	72.45	103.80±12.72	3	98.00±3.45
**I195$^{45.52}$M**	3.05±1.57	1.47	89.43±6.05	3	97.53±1.21
**Y205$^{5.35}$R**	0.34±1.12	0.16	106.20±1.45	3	92.69 ± 2.12
**F294$^{7.35}$Y**	0.99±1.30	0.48	103.10±3.86	3	98.18± 1.38
**D295$^{7.36}$F**	1.39±1.95	0.67	75.57±7.43	3	101.00± 2.09
**SSTR3-WT**	63.82±1.33	1	36.72±11.65	5	100
**N101$^{2.64}$V**	83.73±1.31	1.31	90.71±7.93	3	100.50± 1.79
**H192$^{45.51}$T**	58.10±1.43	0.91	108.40±8.99	3	96.19± 1.76
**M193$^{45.52}$I**	184.40±6.02	2.89	38.77±17.39	3	97.57± 6.28
**F296$^{7.35}$D**	309.00±6.30	4.84	65.55±6.40	3	101.90± 2.86

Supplementary Table 5. Summary of octreotide induced cAMP inhibition assays of SSTRs. Related to Fig. 4 and Supplementary Fig. 7.

Mutation	EC_{50} (nM)	Fold of WT	E_{max} (%WT SSTR2)	n	Expression level (%WT)
**SSTR2-WT**	0.21±1.35	1.00	100	6	100
**V103$^{2.64}$N**	0.23±1.38	1.10	94.00±3.65	3	101.30± 0.69
**T194$^{45.51}$H**	18.73±1.55	89.19	88.27±7.62	3	98.00±3.45
**SSTR3-WT**	30.14±1.49	1	99.85±8.21	5	100
**N101$^{2.64}$V**	1.33±1.23	0.04	120.30±3.57	3	100.50± 1.79
**H192$^{45.51}$T**	21.04±1.73	0.70	92.39±10.25	3	96.19± 1.76

Supplementary Table 6. Summary of paltusotine induced Bystander BRET assays of SSTR2 and mutation. Related to Supplementary Fig. 9.

Mutation	EC_{50} (nM)	Fold of WT	E_{max} (%WT SSTR2)	n	Expression level (%WT)
**SSTR2-WT**	122.30±1.31	1.00	100	6	100
**R140$^{3.50}$A**	7.80±1.47	0.10	50.92±2.01	3	100.57± 1.86
**W269$^{6.48}$A**	130.00±1.65	1.06	27.20±2.61	3	91.28±0.76
**Y312$^{7.53}$A**	8.13±1.64	0.20	41.34±3.00	3	97.22±2.40
**P220$^{5.50}$A**	ND	ND	ND	3	103.80±2.36
**I130$^{3.40}$A**	ND	ND	ND	3	103.00±3.81
**F265$^{6.44}$A**	ND	ND	ND	3	117.80±3.62
**N276$^{6.55}$A**	145.20±1.11	1.19	80.98±1.71	5	114.30±2.22
**I284^{ECL3}A**	89.84±1.28	0.73	106.50±6.25	3	114.20 ±9.70
**K291$^{7.32}$A**	89.38±1.33	0.73	86.76±5.01	3	114.30±2.22
**F294$^{7.35}$A**	2094.00±1.18	17.12	95.10±4.53	3	96.62±3.10

ND means not detected. "–" means no measurement.

Supplementary Table 7. Summary of octreotide induced Bystander BRET assays of SSTR2 and mutation. Related to Supplementary Fig. 9.

Mutation	EC_{50} (nM)	Fold of WT	E_{max} (%WT SSTR2)	n	Expression level (%WT)
**SSTR2-WT**	1.56±1.43	1.00	100	6	100
**R140$^{3.50}$A**	5.78±1.15	3.70	48.39±0.93	2	100.57± 1.86
**W269$^{6.48}$A**	0.43±5.73	0.28	14.52±3.19	3	91.28±0.76
**Y312$^{7.53}$A**	10.20±1.20	6.54	45.64±1.21	2	97.22±2.40
**P220$^{5.50}$A**	949.50±1.78	608.66	20.27±8.01	3	103.80±2.36
**I130$^{3.40}$A**	233.60±1.36	149.36	68.15±4.05	3	103.00±3.81
**F265$^{6.44}$A**	283.40±1.47	181.67	56.12±4.14	3	117.80±3.62
**N276$^{6.55}$A**	ND	ND	ND	3	114.30±2.22
**I284^{ECL3}A**	276.10±1.29	176.99	94.85±4.62	3	114.20 ±9.70
**K291$^{7.32}$A**	1611.00±1.46	976.36	68.05±8.22	3	114.30±2.22
**F294$^{7.35}$A**	ND	ND	ND	3	96.62±3.1

ND means not detected. "–" means no measurement.

a **b**

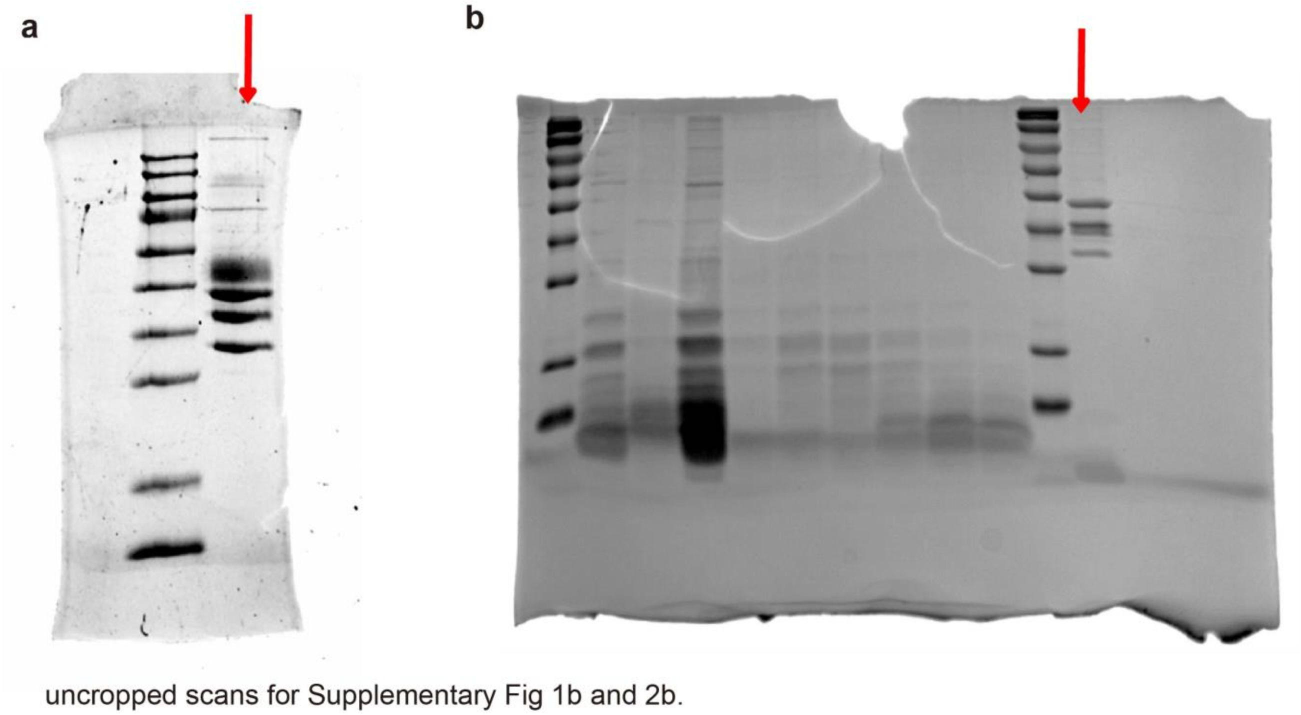

uncropped scans for Supplementary Fig 1b and 2b.
