# Supplementary material for: Prospect of acromegaly therapy: molecular mechanism of clinical drugs octreotide and paltusotine
Source: Nat Commun. 2023 Feb 21;14:962. doi: 10.1038/s41467-023-36673-z (PMC9944328; doi:10.1038/s41467-023-36673-z)
Supplement: Supplementary file 3 — Reporting Summary [file 41467_2023_36673_MOESM3_ESM.pdf]

nature portfolio

Corresponding author(s): Zhenhua Shao; Xiawei We; Wei Yan; Renjie Chai

Last updated by author(s): Jan 27, 2023

Reporting Summary

Nature Portfolio wishes to improve the reproducibility of the work that we publish. This form provides structure for consistency and transparency in reporting. For further information on Nature Portfolio policies, see our Editorial Policies and the Editorial Policy Checklist.

Statistics

For all statistical analyses, confirm that the following items are present in the figure legend, table legend, main text, or Methods section.

n/a	Confirmed	
☐	☒	The exact sample size (*n*) for each experimental group/condition, given as a discrete number and unit of measurement
☐	☒	A statement on whether measurements were taken from distinct samples or whether the same sample was measured repeatedly
☐	☒	The statistical test(s) used AND whether they are one- or two-sided<br>*Only common tests should be described solely by name; describe more complex techniques in the Methods section.*
☒	☐	A description of all covariates tested
☒	☐	A description of any assumptions or corrections, such as tests of normality and adjustment for multiple comparisons
☐	☒	A full description of the statistical parameters including central tendency (e.g. means) or other basic estimates (e.g. regression coefficient) AND variation (e.g. standard deviation) or associated estimates of uncertainty (e.g. confidence intervals)
☐	☒	For null hypothesis testing, the test statistic (e.g. *F*, *t*, *r*) with confidence intervals, effect sizes, degrees of freedom and *P* value noted<br>*Give P values as exact values whenever suitable.*
☒	☐	For Bayesian analysis, information on the choice of priors and Markov chain Monte Carlo settings
☒	☐	For hierarchical and complex designs, identification of the appropriate level for tests and full reporting of outcomes
☒	☐	Estimates of effect sizes (e.g. Cohen's *d*, Pearson's *r*), indicating how they were calculated

Our web collection on statistics for biologists contains articles on many of the points above.

Software and code

Policy information about availability of computer code

Data collection	Cryo-EM data collection were performed on a Titan Krios at 300 kV accelerating voltage in Southern University of Science and Technology Cryo-EM Center (ShenZheng, China) and SKLB West China Cryo-EM Center, Sichuan University (Chengdu, China).The confocal fluorescence images were aquired by Zeiss LSM 880 microscope with the ZEN imaging software (Zeiss). The flow cytometry was acquired by a Novocyte flow cytometer (ACEA Biosciences, China).
Data analysis	Cryo-EM micrographs were recorded using a Gatan K2 and K3 Summit detector using the SerialEM and EPU software. The structures were solved and refined using the Phenix 1.19, Coot 0.8.9.2. MotionCor2, GCTF, RRELION 3.0/4.0-beta, UCSF ChimeraX1.1, PyMOL V2.5.1 package were also used in this study. Signaling assays were analyzed using Graphpad Prism 7. The flow cytometry analysis was analysed with NovoExpress TM software.

For manuscripts utilizing custom algorithms or software that are central to the research but not yet described in published literature, software must be made available to editors and reviewers. We strongly encourage code deposition in a community repository (e.g. GitHub). See the Nature Portfolio guidelines for submitting code & software for further information.

Data

Policy information about availability of data

All manuscripts must include a data availability statement. This statement should provide the following information, where applicable:

- Accession codes, unique identifiers, or web links for publicly available datasets
- A description of any restrictions on data availability
- For clinical datasets or third party data, please ensure that the statement adheres to our policy

The cryo-EM density maps and structural data of SSTR2/octreotide (EMDB ID: EMD-33710 [https://www.ebi.ac.uk/pdbe/entry/emdb/EMD-33710]; PDB ID: 7YAE [http://doi.org/10.2210/pdb7YAE/pdb]) and SSTR2/paltusotine (EMDB ID: EMD-33708 [https://www.ebi.ac.uk/pdbe/entry/emdb/EMD-33708]; PDB ID: 7YAC [http://doi.org/10.2210/pdb7YAC/pdb]) are deposited in EMDB and PDB. The PDB codes and hyperlinks used in our structural alignment were listed below: AT1R-TRV023 (PDB code: 6OS1 [http://doi.org/10.2210/pdb6OS1/pdb]); μOR-Bu72 (PDB code: 5C1M [http://doi.org/10.2210/pdb5C1M/pdb]) or FH210 (PDB code: 7SCG [http://doi.org/10.2210/pdb7SCG/pdb]); the inactive SSTR2 (PDB code: 7UL5, colored in wheat [http://doi.org/10.2210/pdb7UL5/pdb]); SSTR2-SST14(PDB code: 7T10 [http://doi.org/10.2210/pdb7T10/pdb]). Other data are available in the main text or the supplementary materials.

Human research participants

Policy information about studies involving human research participants and Sex and Gender in Research.

Reporting on sex and gender	N/A
Population characteristics	N/A
Recruitment	N/A
Ethics oversight	N/A

Note that full information on the approval of the study protocol must also be provided in the manuscript.

Field-specific reporting

Please select the one below that is the best fit for your research. If you are not sure, read the appropriate sections before making your selection.

☒ Life sciences ☐ Behavioural & social sciences ☐ Ecological, evolutionary & environmental sciences

For a reference copy of the document with all sections, see nature.com/documents/nr-reporting-summary-flat.pdf

Life sciences study design

All studies must disclose on these points even when the disclosure is negative.

Sample size	For cryo-EM data, sample sizes were determined by availability of microscope. Cryo-EM data was collected until we were able to refine a high-resolution structure that allowed us to obtain a high-resolution reconstruction within the confines of limited microscope time.
Data exclusions	No data was excluded from the analyses.
Replication	Protein samples were purified from different purification batch and were performed for cryo-EM. Our primary data are cryo-EM structures that were calculated according to standard procedures and the cryo-EM data were calculated in over 6159 and 4746 individual movies. The biochemical experiments in this study have been repeated by greater than or equal to three independent experiments, and those finding are reliably reproduced.
Randomization	Randomization is not relevant to this study. Our structures were calculated according to standard procedures with freely available software and does not need randomization
Blinding	Our experiments were all biochemical studies, no blinding was used or necessary during data collection or analysis, as this is not a hypothesis driven study and also because of the nature of structural biology, however appropriate controls are included. As above, Our primary data are cryo-EM structure that were calculated according to standard procedures with freely available software and did not require blinding.

Reporting for specific materials, systems and methods

We require information from authors about some types of materials, experimental systems and methods used in many studies. Here, indicate whether each material, system or method listed is relevant to your study. If you are not sure if a list item applies to your research, read the appropriate section before selecting a response.

Materials & experimental systems

n/a	Involved in the study	
☐	☒	Antibodies
☐	☒	Eukaryotic cell lines
☒	☐	Palaeontology and archaeology
☒	☐	Animals and other organisms
☒	☐	Clinical data
☒	☐	Dual use research of concern

Methods

n/a	Involved in the study	
☒	☐	ChIP-seq
☐	☒	Flow cytometry
☒	☐	MRI-based neuroimaging

Antibodies

Antibodies used	Antibodies used: anti-FLAG HRP-conjugated monoclonal antibody (Proteintech, Catalog No. 66008-4-Ig, Clone No. 8H6A10). 1:1000 diluted with PBS containing 5% BSA
Validation	The Anti-FLAG HRP-conjugated mouse monoclonal antibody is well characterized and was applied according to data sheet information details.(As supplied in the manufacturer's website, 66008-4-Ig targets DYKDDDDK tag in WB, RIP, IP, IF, CoIP, ChIP, ELISA applications and shows reactivity with recombinant protein samples.) https://www.ptgcn.com/products/Flag-tag-Antibody-66008-4-Ig.htm

Eukaryotic cell lines

Policy information about cell lines and Sex and Gender in Research

Cell line source(s)	Sf9 cells, Expression systems, Cat. 94011S. HEK293 cells, ATCC, CRL-1573. GH3 cells, ATCC, CCL-82.1.
Authentication	The cell lines are maintained by the supplier and verified by short tandem repeat (STR) profiling method. The cell used in this study are all from low-passage cell lines. Their morphology and growth curve analysis are further checked using microscope and cell counter respectively by investigators in this study.
Mycoplasma contamination	The cell lines are tested by manufacturer for contamination and are negative for mycoplasma contamination.
Commonly misidentified lines (See ICLAC register)	No commonly misidentified cell lines were used.

Flow Cytometry

Plots

Confirm that:

☒ The axis labels state the marker and fluorochrome used (e.g. CD4-FITC).

☒ The axis scales are clearly visible. Include numbers along axes only for bottom left plot of group (a 'group' is an analysis of identical markers).

☒ All plots are contour plots with outliers or pseudocolor plots.

☒ A numerical value for number of cells or percentage (with statistics) is provided.

Methodology

Sample preparation	Cell apoptosis assay<br>The rat pituitary GH3 cells were pre-seeded in 12-well plates. Octreotide or paltusotine were added to each well at the indicated concentrations and incubated for 48 hours.Then, the cells were collected for the apoptosis assay using the Annexin V-FITC/PI apoptosis detection kit (BD Biosciences).The cells were washed with cold PBS and resuspended in 1 × binding buffer. Then, 5 μl of Annexin V-FITC was added and incubated for 10 min and stained with 5 μl of PI for 5 min in the dark at room temperature. All samples were acquired and analyzed by the flow cytometry.
Instrument	Flow cytometric data were collected using NovoCyte (ACEA bioscience. Inc).
Software	NovoExpress 1.4.1.
Cell population abundance	Cell lines (100%).
Gating strategy	FSC-H vs. SSC-H - gated on cells, SSC-H vs. SSC-A - gated on singlets. Cells in Annexin V+/PI– and Annexin V+/PI+ gates were both considered as the apoptotic cells.

☒ Tick this box to confirm that a figure exemplifying the gating strategy is provided in the Supplementary Information.
